# Environmental drivers for cheaters of arbuscular mycorrhizal symbiosis in tropical rainforests

**DOI:** 10.1111/nph.15876

**Published:** 2019-05-30

**Authors:** Sofia I. F. Gomes, Peter M. van Bodegom, Vincent S. F. T. Merckx, NadejdaA. Soudzilovskaia

**Affiliations:** ^1^ Institute of Environmental Sciences Leiden University 2333 CC Leiden the Netherlands; ^2^ Understanding Evolution Group Naturalis Biodiversity Center 2332 AA Leiden the Netherlands; ^3^ Department of Evolutionary and Population Biology Institute for Biodiversity and Ecosystem Dynamics University of Amsterdam Amsterdam the Netherlands

**Keywords:** arbuscular mycorrhizal fungi, cheating, mycoheterotrophy, nitrate, potassium, soil

## Abstract

- Hundreds of nonphotosynthetic mycoheterotrophic plant species cheat the arbuscular mycorrhizal symbiosis. Their patchy local occurrence suggests constraints by biotic and abiotic factors, among which the role of soil chemistry and nutrient status has not been investigated.
- Here, we examine the edaphic drivers predicting the local‐scale distribution of mycoheterotrophic plants in two lowland rainforests in South America. We compared soil chemistry and nutrient status in plots where mycoheterotrophic plants were present with those without these plants.
- Soil pH, soil nitrate, and the interaction between soil potassium and nitrate concentrations were the best predictors for the occurrence of mycoheterotrophic plants in these tropical rainforests. Mycoheterotrophic plant occurrences decreased with a rise in each of these predictors. This indicates that these plants are associated with low‐fertility patches. Such low‐fertility conditions coincide with conditions that potentially favour a weak mutualism between plants and arbuscular mycorrhizal fungi according to the trade balance model.
- Our study points out which soil properties favour the cheating of arbuscular mycorrhizal networks in tropical forests. The patchy occurrence of mycoheterotrophic plants suggests that local soil heterogeneity causes the stability of arbuscular mycorrhizal networks to vary at a very small scale.

## Introduction

Mycorrhizal symbiosis is one of the most widespread interactions on Earth (van der Heijden *et al*., 2015). Typically, it is a mutually beneficial interaction in which plants transfer photosynthesised carbon to their mycorrhizal fungal partners, which in turn facilitate the uptake of mineral nutrients from the soil, enhancing plant nutrition (Smith & Read, 2008). Symbiosis is therefore extremely important in soils of low nutrient availability or where the distribution of nutrients is heterogeneous (Cavagnaro *et al*., 2005). Yet, mycoheterotrophic plants have evolved a strategy in which the carbon flux is reversed from their fungal partners to themselves so that the plants depend exclusively on their mycorrhizal partners to obtain carbohydrates (Leake, 1994). It remains to be investigated whether these plants provide any benefit to their associated fungi, such as vitamins or protection, and therefore reciprocate Alternatively, they may subvert the ‘biological market’ established between plants and mycorrhizal fungi, where plants trade carbohydrates for soil nutrients with their mycorrhizal partners (Selosse & Rousset, 2011). There are over 500 fully mycoheterotrophic plant species, of which about half the total number is associated with arbuscular mycorrhizal (AM) fungi (Merckx, 2013). As these plants require the continuous presence of an established mycorrhizal network to support their carbon demands during the entire life cycle, ultimately relying on the surrounding photosynthetic plants, mycoheterotrophy can be regarded as a mechanism enabling cheating of mycorrhizal symbiosis (van der Heijden & Walder, 2016).

Many species of mycoheterotrophic plants have remarkably widespread distributions, yet at a local scale their distribution is often highly scattered (Cheek & Williams, 1999; Bergman *et al*., 2006; Merckx *et al*., 2013; Yamato *et al*., 2016). The patchy occurrence of these plants suggests that, besides the need to fulfil general global scale requirements, such as appropriate soil water content (Maas *et al*., 1986; Cheek & Williams, 1999) or shade conditions (Leake, 1994; Cheek & Williams, 1999; Bidartondo *et al*., 2004) within particular forest types (Gomes *et al*., 2019), the presence of mycoheterotrophic plants is constrained by particular local‐scale factors. Due to the reliance of mycoheterotrophic plants on mycorrhizal networks, both biotic (interactions with their fungi) and abiotic (soil conditions) factors can potentially contribute to their occurrence at a local scale. Previous studies have shown highly species‐specific interactions between these plants and their fungal partners from a local to a global scale (Yamato *et al*., 2016; Gomes *et al*., 2017a; Renny *et al*., 2017), although the degree of specificity may vary (Courty *et al*., 2011; Gomes *et al*., 2017b). This situation could indicate that the occurrence of their fungal associates may determine the distribution of mycoheterotrophic plants (Bougoure *et al*., 2009; McCormick *et al*., 2009; Yamato *et al*., 2016). However, Merckx *et al*. (2017), suggesting that the distribution of AM fungi does not drive the distribution of highly specialised mycoheterotrophic plants in the genus *Thismia*, as their specific fungal associates were found to occur beyond the range of the plants’ distribution. Also, for mycoheterotrophic plants associated with ectomycorrhizal fungi, it has been shown that mycoheterotrophs are not present in all instances in which their fungal partners are available (Ogura‐Tsujita & Yukawa, 2008; Waterman *et al*., 2011; Davis *et al*., 2015). Hence, the presence of specific fungi alone is probably not sufficient to explain why mycoheterotrophs establish in particular patches of soil and avoid others. Neither is the presence of specific autotrophic green hosts, as AM mycoheterotrophs have been reported to occur in forests dominated by diverse tree species across continents (Merckx *et al*., 2013 and references therein). Moreover, soil nutrient availability may have an impact on the occurrence of mycoheterotrophic plants by affecting these directly or indirectly via the AM networks upon which these plants rely. Studies that examine which soil characteristics influence the occurrence of mycoheterotrophic plants at a local scale, either directly or indirectly, are lacking. Here, we examined which soil properties are associated with the patchy presence of AM mycoheterotrophic plants at the local scale. We conducted our study in two tropical rainforest regions differing in, among other factors, soil fertility, to assess the generality of the relationships.

## Materials and Methods

### Study area

Mycoheterotrophic plants are ephemeral, and their flowering periods are quite short (Leake, 1994). This situation indicates that there is probably a temporal and seasonal variation in the factors that drive the occurrence of these plants. This study took place at the beginning of the wet season, and coincided with the time of year when mycoheterotrophs have been recorded to flower (Cheek & Williams, 1999), reflecting the most favourable conditions to find them in the field. We sampled two forest regions in Colombia, where mycoheterotrophic plant species are known to occur. We spent 5 d sampling in each region. The first region consisted of wet tropical lowland forest on *terra firme*, part of the Amazon rainforest near Leticia (‘Amazon’; 4°00′30″S 70°06′12″W). The second region consisted of wet tropical coastal forest on *terra firme*, part of the Chocó rainforest, near Buenaventura (‘coast’; 3°55′24″N 77°18′56″W).

Large‐scale patterns of soil properties do not necessarily reflect the high heterogeneous profiles of soil at a local scale, therefore we opted for a paired plot sampling strategy – in each of the regions – where a ‘positive’ plot with mycoheterotrophic plants was simultaneously selected alongside with a nearby ‘negative’ plot without visible mycoheterotrophic plants. Because mycoheterotrophic plants without flowers can be overseen due to their small habit and lack of vegetative structures, it is possible that these plants were still present in the negative plots, therefore we carefully searched for these by removing the leaf litter in the ‘negative’ plots. Through this design we were able to identify the effects of specific local differences in soil properties on the patchy occurrences of mycoheterotrophy, within the presumable large‐scale variation in soil parameters among regions. Furthermore, it is possible that particular autotrophic plant species have a certain impact in shaping soil properties; this could also contribute to differences between plots. In this case, the impact of functional plant types is reflected in the soil properties, including their potential associations to particular AM fungi, which are accounted for by using the paired design of our study.

We established 16 pairs of plots of 4 × 4 m in the two forests that represented the spatial extent to which populations of mycoheterotrophic plants often occur, based on > 10 yr of personal observations in the field (by VSFT Merckx, and personal communication with P. Maas), and previous studies (Gomes *et al*., 2017b). We delimited five pairs of plots in the Amazon and 11 on the coast. Positive and negative plots were 5–10 m apart, this distance was close enough to avoid a change in substrate conditions once mycoheterotrophic plants were no longer observed. Pairs of plots were separated by at least 30 m to ensure sufficient distance between paired plots. The number of mycoheterotrophic plants in the positive plots varied between 1 and 22 individuals, and we found up to six species per plot (Supporting Information Table S1). Within each plot, we randomly collected six soil cores, and combined them into a 250 g composite sample per plot. The soil in both regions had clay texture. Soil cores were taken in the shallow top layer of the soil (0–5 cm depth) because we were interested in the chemical properties and nutrient abundance in the soil layer where the roots of the mycoheterotrophic plants are found. Big stones and roots were removed from the samples. The soil was homogenised and preserved on ice immediately after collection and during transportation to the laboratory for further processing.

While we recognise that biotic interactions including the abundance of associated fungi can play an important role on plant's occurrence patterns (Chagnon & Bradley, 2013), methods to quantify biomass of particular AM taxa while excluding others – to target the preferred AM fungi associated with mycoheterotrophs – are currently unavailable. Furthermore, previous studies have shown that the mere presence of the preferred AM fungi does not guarantee the presence of a mycoheterotroph (Ogura‐Tsujita & Yukawa, 2008; Waterman *et al*., 2011; Davis *et al*., 2015; Merckx *et al*., 2017). Therefore, we did not include biotic interactions in our analysis.

### Soil chemistry and nutrient analyses

Soil chemical and nutrient properties were assessed for all 32 plots. Each sample was analysed for soil pH. Total amounts of nitrogen (N_tot_) and phosphorus (P_tot_) were estimated using the Kjeldahl method (Bremner, 1960). The available nitrogen (NH_4_^+^ and NO_3_^−^) in the soil was determined using spectrophotometry and 1 N potassium chloride (Maynard & Kalra, 1993). The available phosphorus (P_av_) was extracted using a Bray II solution (Murphy & Riley, 1962). Exchangeable bases (Na, K, Ca and Mg) were measured using the ammonium acetate method (Hanway & Heidel, 1952) and determined using atomic absorption spectrometry. The available micronutrients (Cu, Zn, Mn and Fe), available boron (B), sulfur (S), aluminium (Al), cation exchange capacity (CEC), and soil moisture content were determined according to Carter & Gregorich (2008). Organic matter (OM) content in the soil was determined according to Walkley & Black, 1934. All analyses were performed using the Centro Internacional de Agricultura Tropical in Colombia. Total soil C and N (on air‐dried soil), and abundance of δ^13^C and δ^15^N were analysed at UC Davis (University of California, Davis). To evaluate the influence of nutrient stoichiometry on soil processes, we calculated the N : P, N : K, C : P and C : N ratios.

### Data analysis

We tested for differences in overall soil composition among positive and negative plots across both regions – that might affect the relationships to the local patchy occurrence of mycohetereotrophic plants – using a one‐way permutational multivariate analysis of variance (perMANOVA with 999 permutations). We tested for homogeneity of dispersion among groups before performing the perMANOVA and confirmed the assumption of homogeneous dispersion among regions (*P* = 0.753), and between negative and positive plots within the Amazon (*P* = 0.198) and the coast (*P* = 0.873). We visualised these differences through principal component analysis (PCA).

Given the heterogeneity in soil properties at the regional scale, average concentrations of nutrients in the soil in positive and negative plots can disguise the true effect of specific soil properties on the local selection of mycoheterotrophic occurrences. Our paired plot design allows a detailed local‐scale analysis, focusing on local differences. Therefore, we calculated the difference in the soil parameter values within each pair of negative and positive plots, which hereafter we refer to as delta (∆). A negative delta indicated that a specific parameter was lower in plots where mycoheterotrophic plants were absent, and a positive delta indicated that the parameter was lower in the plot where these plants were present. We tested whether there were significant differences across all deltas of the soil properties among regions using perMANOVA (homogeneity of dispersion: *P* = 0.713). We examined whether the delta of individual soil properties varied across regions using ANOVAs with ‘Region’ as factor, using the general linear hypothesis test (glht) function of R package lsmeans. Accounting for region in the analysis, allows assessing whether soil properties associated to the presence of mycoheterotrophic plants differ between regions. These delta values do not represent the actual concentrations under which plants are influenced, but they allow us to better quantify the effects of local‐scale variations in soil nutrients. Furthermore, we examined whether the actual values of the soil properties varied between positive and negative plots within each region using ANOVAs with subsequent Tukey's Honest Significant Difference test for correction of the *P*‐values.

To assess which combination of soil properties was most strongly related to the distribution of mycoheterotrophic plants, we selected all soil properties that were significantly different between positive and negative plots. Using the actual measurements from the soil properties in each positive and negative plot, each soil property was standardised to mean = 0 and SD = 1 to avoid scaling variance issues due to different measurement scales. With the soil properties, we built generalised mixed‐effects models (GLMMs) with ‘region’ and ‘plot’ as random effect terms to account for the overall soil properties differences among regions, and our paired plots design, respectively. To better understand the drivers for both the occurrence and abundance of mycoheterotrophic plants, we built two models with the presence/absence, and the density of mycoheterotrophic plants, respectively, as dependent variable in each of the models.

Model selection was performed for each model separately by adding terms, including interactions between variables, and selecting the terms that gave the greatest improvement to the model likelihood, as assessed using the lowest Bayesian Information Criteria (BIC; Aho *et al*., 2014). The variables included in the final model were retained if they were significant, and had a variance inflation factor (VIF) < 4 (Zuur *et al*., 2010) and showed a Pearson correlation with all other modelled predictors < |0.70| (Dormann *et al*., 2013). The coefficient of determination (*R* ^2^) of the GLMMs was calculated based on Nakagawa & Schielzeth (2013).

All analyses were performed in R 3.4.1 (R Core Team, 2016), using the packages ‘lme4’, ‘multcomp’, ‘r2glmm’, ‘vegan’, ‘lsmeans’.

## Results

### Soil characteristics

**Figure 1:**
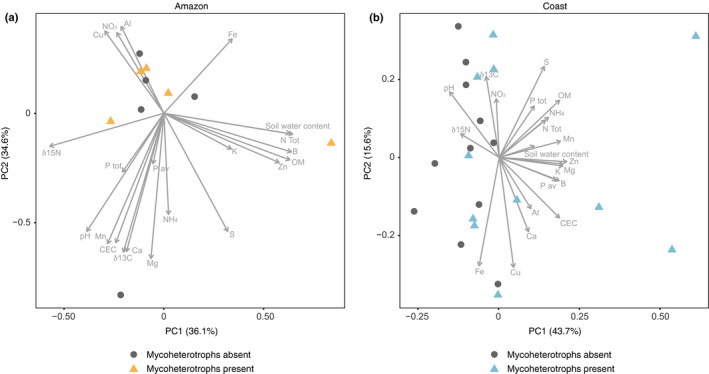
Principal component (PC) analysis of the soil properties in the positive plots (triangles) and negative plots (circles) present in (a) the Amazon and (b) the coast. Length of the arrows represents the relative importance of individual properties in explaining the overall pattern.

**Figure 2:**
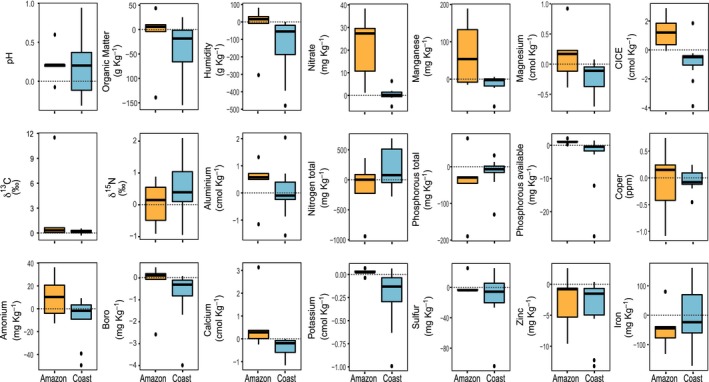
Box and whisker plots representing the variation of soil properties between the negative and positive plots in the Amazon (yellow) and the coast (blue). Positive values indicate higher availability of a soil property in the negative plots, while negative values indicate a higher availability in the positive plots.

We obtained 21 soil parameters from the soil analyses (Table S2). Overall, soil characteristics were significantly different between the two regions (*F* = 28.338, *R* ^2^ = 0.49, *P *=* *0.001; Fig. S1). Across all soil parameters, there was no general significant difference between positive and negative plots in the Amazon (*F* = 0.738, *R* ^2^ = 0.08, *P *=* *0.627; Fig. 1a), but there was a difference in the coast (*F* = 3.079, *R* ^2^ = 0.13, *P *=* *0.044; Fig. 1b). In addition, across all deltas of soil parameters there was a tendency for more positive delta values in the Amazon than in the coast (*F* = 0.266, *R* ^2^ = 0.14, *P *=* *0.051; Fig. 2). When considering each soil property individually, in the Amazon region, the availability of NO_3_, P_av_, CEC and pH was significantly lower (Table S2) in the positive plots compared to the respective negative plots. These are different soil properties than those varying most within the Amazon region, being OM, soil moisture content, N_tot_, ^15^N, B and Zn (Fig. 1a). This result indicates that the variation in soil properties at the regional scale in the Amazon was different from the variation in soil properties at the local scale. At the coast, positive plots had significantly higher availability of OM, soil moisture content, CEC, N_tot_, and positive ions such as K, Ca, Mg, B, Zn; and lower availability of ^15^N (Table S2), which corresponded to the same soil properties that had more variation within this region (Fig. 1b). This indicates that the variation in soil properties at the local scale is similar to those at the regional scale in the coast. At the local scale, the deltas of soil showed different trends within each region, suggesting that the local selection of mycoheterotrophic plants by soil properties, leading to the local patchy distribution of mycoheterotrophic plants, differs among the regions (Fig. 2; Table S3). We selected those soil properties that were significantly different between positive and negative plots in both regions (Table S2) to build the generalised mixed‐effects models.

### Model selection

**Figure 3:**
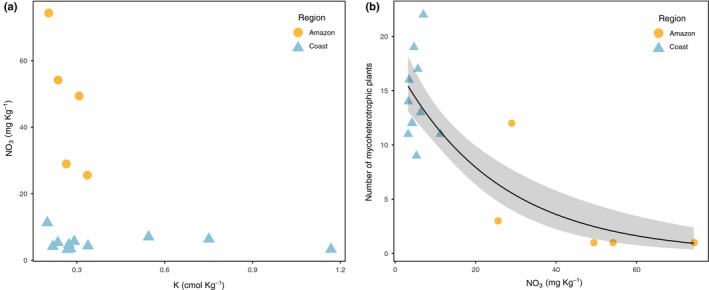
Relationships between (a) the actual concentration of NO_3_ vs K when in positive plots, showing the nature of interaction between the two nutrients. When K is the lowest, NO_3_ varies, and when NO_3_ is available in the lowest concentrations, K varies; and (b) the number of mycoheterotrophic plants observed in the positive 4 × 4 m plots and the concentration of NO_3_. The solid line represents the observed trend with the 95% confidence interval (grey area).

The best model for the occurrence of mycoheterotrophic plants showed a significant effect of NO_3_, pH and the interaction between NO_3_ and K (GLMM: *R* ^2^ = 0.64, BIC = 43.8; Table 1). The nature of the interaction between NO_3_ and K is explained by NO_3_ varying while K is constant in the Amazon, and of K varying while NO_3_ is constant in the coast among negative and positive plots (Fig. 3a; Table S3). The second best model showed a significant effect of soil moisture content and pH and the interaction between soil moisture content and OM (GLMM: *R* ^2^ = 0.54, BIC = 49.7; Table 1). OM was highly correlated with K (Pearson correlation: *R* ^2^ = 0.67) and Zn (Pearson correlation: *R* ^2^ = 0.72), and K correlated with Zn (Pearson correlation: *R* ^2^ = 0.74), not allowing to separate their impacts. Therefore, these parameters were not included in the same model.

**Table 1:** Outcomes of the Generalised Linear Mixed Effect modelling aimed to explain the occurrence of mycoheterotrophic plants

Model	Terms	Coefficient	SE	*z* value	*P*‐value
1	Intercept	1.038	0.710	1.463	0.144
	NO_3_	−3.432	1.347	−2.548	0.011
	pH	−1.405	0.820	−1.713	0.087
	NO_3_ : K	−11.165	4.264	−2.618	0.009
2	Intercept	−0.474	0.614	−0.772	0.440
	Soil moisture content	5.052	2.377	2.126	0.034
	pH	−2.157	1.137	−1.897	0.058
	pH : OM	4.538	2.226	2.038	0.042

Model 1 is the best model; 2 is the best alternative model (ΔBIC = 5.9).

When density of mycoheterotrophic plants was evaluated instead of occurrence, we obtained two undistinguishable best models. The first model showed a significant effect of soil moisture content, pH and the interaction between NO_3_ and K (GLMM: *R* ^2^ = 0.11, BIC = 247.1; Table 2). The other best model showed a significant effect of soil moisture content and pH and the interaction between pH and OM (GLMM: *R* ^2^ = 0.11, BIC = 244.9; Table 2).

**Table 2:** Outcomes of the Generalised Linear Mixed Effect modelling aimed to explain the density of mycoheterotrophic plants

Model	Terms	Coefficient	SE	*z* value	*P*‐value
1	Intercept	−0.538	1.240	−0.434	0.665
	Soil moisture content	2.440	0.974	2.505	0.012
	pH	−1.377	0.417	−3.306	0.001
	NO_3_ : K	−2.634	1.131	−2.330	0.020
2	Intercept	0.674	0.69	0.996	0.334
	Soil moisture content	3.023	0.746	4.050	5.12e^−05^
	pH	−1.976	0.391	−5.056	4.29e^−07^
	pH : OM	1.108	0.360	3.080	0.002

Models 1 and 2 are not significantly different (ΔBIC = 2.2).

The number of mycoheterotrophic plants decreased with the increasing difference in concentration of ΔNO_3_ or NO_3_, corresponding to an increase in total concentration of NO_3_ (Pearson correlation between ΔNO_3_ and NO_3_: *R* ^2^ = 0.77; Fig. 3b).

## Discussion

In this study, we compared soil characteristics of paired plots with and without flowering mycoheterotrophic plants to infer local‐scale drivers that influenced the occurrence and abundance of plant cheaters in the AM symbiosis in tropical rainforests. The Amazon and coast sites had overall different soil properties, leading to distinct soil properties that affected the presence of mycoheterotrophic plants at the regional scale. These regional differences in soil properties and associated regional preferences of mycoheterotrophs do not necessarily reflect local‐scale preferences. When the datasets of the Amazon and coast regions were analysed together, to better understand the local‐scale drivers for the occurrence of mycoheterotrophic plants, we found that the strongest local edaphic predictors of mycoheterotrophic species occurrences involved the interaction between NO_3_ and K, and the individual effects of NO_3_ and pH. Concerning the density of mycoheterotrophic plants individuals, we found that soil moisture content and pH together with an interaction between either NO_3_ and K, or pH and OM, best explained the densities found in the sampled plots. The model predicting the density of mycoheterotrophic plants explained less variance than the model predicting their occurrence, suggesting that other predictors, not accounted in the present study, may be important to determine the density of these plants. It also showed that soil moisture content appeared to influence mycoheterotrophic plants’ occurrence, but to a lesser extent than soil fertility at the local scale overall the two regions in this study. Soil moisture has been hypothesised to be the main limiting factor to the occurrence of these plants at the global scale, due to their sensitivity to desiccation (Leake, 1994; Klooster & Culley, 2009), but our study showed that mycoheterotrophic plants had a stronger selection for other soil conditions at the local scale. Due to the high correlation between K and OM and K and Zn, the effect of K in both models may also partly resemble effects through OM and/or Zn. Consistently the same interacting terms, between NO_3_ and K, were selected in models for both the occurrence and density of mycoheterotrophic plants.

The interaction between soil NO_3_ and K is well known to mediate crop responses to fertilisation. Crop response to added nitrogen fertilisers decreases when the exchangeable potassium content of a soil is below an optimal level, because plants deficient in potassium content are not able to produce proteins despite an abundance of available nitrogen (Ranade‐Malvi, 2011). In addition, several studies have shown a negative impact of nitrogen addition in agriculture systems on the AM symbiosis performance (Kabir *et al*., 1998; Galvez *et al*., 2001; Oehl *et al*., 2004), because an increased availability of nitrogen to plant roots can lead to a reduced allocation of carbon to their AM fungal partners, which can in turn induce phosphorus deficiency due to carbon limitation to the fungi (Olsson *et al*., 2005). Moreover, excessive amounts of N reduce the plant uptake of P, K and other micronutrients (Ranade‐Malvi, 2011). Nutrient stoichiometry in soils has been shown to be crucial in determining the relative availability of nutrients for plant uptake and the stability of the AM symbiosis (Johnson, 2010; Khan *et al*., 2015). The importance of NO_3 _: K stoichiometry revealed using our models suggested that for the occurrence and density of mycoheterotrophic plants, nutrient stoichiometry matters. Next to nutrient stoichiometry, pH was also an important predictor. The pH strongly influences the availability of nutrients in the soil, which in turn also impacts the efficiency of nutrient uptake by plants (Rippy *et al*., 2004), and by the AM fungi directly (see for example Ouzounidou *et al*., 2015), or indirectly through other associated microbes such as bacteria (Svenningsen *et al*., 2018).

Our study showed that the impacts of NO_3_ and K on mycoheterotrophic plant occurrence depend on soil fertility. At conditions of heterogeneous high fertility patches and high soil moisture in the Amazon, mycoheterotrophic plants tend to occur in patches with low NO_3_, probably to avoid high fertility conditions, while there is no selection for K. In the coast, where fertility is lower than in the Amazon, there is no selection for NO_3_ but occurrence of mycoheterotrophs tends to be driven by potassium availability instead. The effect of potassium in the coast may reflect a preference for high OM in this region. potassium and OM were correlated, and OM was significantly higher in positive plots. This implies that habitat conditions at the regional level determined the extent to which particular local drivers are important. While there seems to be a consistent avoidance of higher fertility patches, also reflected in the lower density of plants found with increasing NO_3_ availability (Fig. 3b), it remains unclear how K influences the distribution of these plants. A possible explanation for the overall increased availability and patchiness of K in the coast is the effect of salt spray from the sea that is close by. The uptake of K by photosynthetic plants is enhanced by the association with AM fungi, which require a minimum availability of K in the soil for the stability of the AM symbiosis (Khan *et al*., 2015).

Interestingly, available phosphorus did not relate to local mycoheterotrophic plants’ occurrences in this study, even though we find a trend for plants to select for lower N : P ratios in the positive plots compared with the negative plots (Table S2). This result strongly contrasted to the impacts of phosphorus on plant and microbial communities at regional scales: available phosphorus – together with soil moisture – has been suggested to be the strongest environmental predictor of plant species distributions in tropical forests (Condit *et al*., 2013); and phosphorus is considered to be the main limiting element in tropical forests for microbial processes, including mycorrhizal fungi (Camenzind *et al*., 2017). Moreover, AM fungal diversity is known to be lower when phosphorus availability is very high (Gosling *et al*., 2013). At regional scales, phosphorus has also been shown to play an important role in determining plant and fungal distributions, including those of mycoheterotrophic plants (Sheldrake *et al*., 2017). In a natural fertility gradient across a 65 km forest in Panama, Sheldrake *et al*. (2017) tested the impact of nutrient availability on the occurrence and density of two mycoheterotrophic species of the genus *Voyria,* at the regional scale. Their results suggested that the occurrence of these plants is limited by high phosphorus concentration in the soil, which was further supported by the outcomes of their nutrient addition experiment. In our study, at the local scale, phosphorus did not significantly vary between the positive and negative plots. This finding suggested that there is not a direct selection for particular concentrations of phosphorus at the selected local ecological scale. Instead, our results highlighted that nutrient stoichiometry (as exemplified by the NO_3 _: K interaction) rather than the actual concentration of any nutrient, including phosphorus, drives the local occurrence and density of mycoheterotrophic plants. Hence, our study stressed the fact that regional patterns can be different from local drivers.

Also, the trade balance model (Johnson, 2010) that aims to explain the stability of symbiotic outcomes between plants and AM fungi, stresses the critical role of stoichiometry of soil nutrients compared to the actual abundances of nutrients. Within this framework, a stable relationship is expected when the trade of C‐for‐P is advantageous for both plants and AM fungi. When this stable relationship occurs at high N : P ratios, both AM fungi and plants have easy access to N. Therefore, high N : P ratios may stimulate plants to invest more C to their AM fungi, which in turn provides plants with increased access to P in the soil, resulting in a strong mutualism (Johnson *et al*., 2003). By contrast, at low N : P ratios, plants and AM fungi compete for N, originating less C available in the system, leading to a less balanced or weaker mutualism (Johnson *et al*., 1997). Despite the known importance of P in determining the general occurrence and distribution of plants (Condit *et al*., 2013) and AM fungi (Camenzind *et al*., 2017), and of the N : P ratio for the stability of AM networks (Johnson, 2010), the N : K ratio and not the N : P ratio appeared to be a more relevant predictor for the local distribution of mycoheterotrophic plants. In our study, mycoheterotrophic plants preferred patches with lower N : K ratios by selecting for high K at high fertility conditions, whereas at low‐fertility conditions, they preferred patches with general lower nutrients availability and lower N and P – and to a lesser extent K – but not sufficiently to significantly affect the effect of N : K and K : P ratios (Table S2). In our study, mycoheterotrophic plants seemed to avoid high fertility patches where fungi are prone to parasitise autotrophic plants (high N, high P or K), as suggested by lower N : P or N : K ratios. Following the trade balance model (Johnson, 2010), we hypothesised that mycoheterotrophic plants avoid patches with conditions that coincide with those favouring a strong mutualism between plants and AM fungi (high N, low P or K), preferring conditions that were similar to when the mutualism is weak (low N, low P or K). In conditions leading to commensalism between plants and AM fungi (low N, high P or K), cheating is less likely to occur, as both partners are exchanging limited resources, and therefore it is theoretically more difficult for mycoheterotrophic plants to obtain carbon from these fungi. We believe that this hypothesis deserves further testing.

In conclusion, our study highlighted the scale‐dependent effect of environmental drivers and complemented the current knowledge on the ecological edaphic preferences of mycoheterotrophic plants. Leake (1994) has suggested high soil moisture and deep shaded forests as the preferred habitats for the distribution of mycoheterotrophic plants at the global scale. Sheldrake *et al*. (2017) revealed that phosphorus concentrations limit the occurrence of mycoheterotrophic plants along gradients at the regional scale. Finally, the present study unravelled the importance of nutrient stoichiometry in the soil to explain the patchy occurrence pattern of these plants at the very local scale. Our findings also showed a negative response of the abundance of mycoheterotrophs individuals to an increase in N availability. Furthermore, our results provided empirical support to pose the hypothesis that mycoheterotrophic plants seem to avoid conditions that could favour a strong AM mutualism (high N, low P or K), according to the trade balance model (Johnson, 2010).

## Author contributions

SIFG, PMvB, VSFTM and NAS designed the study. SIFG and VSFTM conducted fieldwork. SIFG conducted the analyses and wrote the first draft of the manuscript. All authors contributed to discussion and to earlier versions of the manuscript.

## Supporting information

Please note: Wiley Blackwell are not responsible for the content or functionality of any Supporting Information supplied by the authors. Any queries (other than missing material) should be directed to the *New Phytologist* Central Office.

**Fig. S1** Principal component analysis of the soil properties in the Amazon and the coast.
**Table S1** Mycoheterotrophic plant species present in the study plots.
**Table S2** Overall soil parameters in the negative and positive plots within the Amazon and coast regions.
**Table S3** Variation of the soil parameters measured in the plots within the Amazon and coast calculated by the difference between negative and positive plots (deltas).
