# Supplementary material for: Environmental drivers for cheaters of arbuscular mycorrhizal symbiosis in tropical rainforests
Source: New Phytol. 2019 May 30;223(3):1575–83. doi: 10.1111/nph.15876 (PMC6771734; doi:10.1111/nph.15876)
Supplement: Supplementary file 1 — Fig. S1 Principal component analysis of the soil properties in the Amazon and the coast. Table S1 Mycoheterotrophic plant species present in the study plots. Table S2 Overall soil parameters in the negative and positive plots within the Amazon and coast regions. Table S3 Variation of the soil parameters measured in the plots within the Amazon and coast calculated by the difference between negative and positive plots (deltas). [file NPH-223-1575-s001.pdf]

***New Phytologist* Supporting Information**

Article title: Environmental drivers for cheaters of arbuscular mycorrhizal symbiosis in tropical rainforests

Authors: Sofia IF Gomes, Peter M van Bodegom, Vincent SFT Merckx, Nadejda A Soudzilovskaia

Article acceptance date: 15 April 2019

The following Supporting Information is available for this article:

Fig. S1: Principal Components Analysis of the soil properties in the Amazon and the Coast.

Table S1: Mycoheterotrophic plant species present in the 16 plots.

Table S2: Overall soil parameters in the negative and positive plots within the Amazon and Coast regions.

Table S3: Variation of the soil parameters measured in the plots within the Amazon and Coast calculated by the difference between negative and positive plots (deltas).

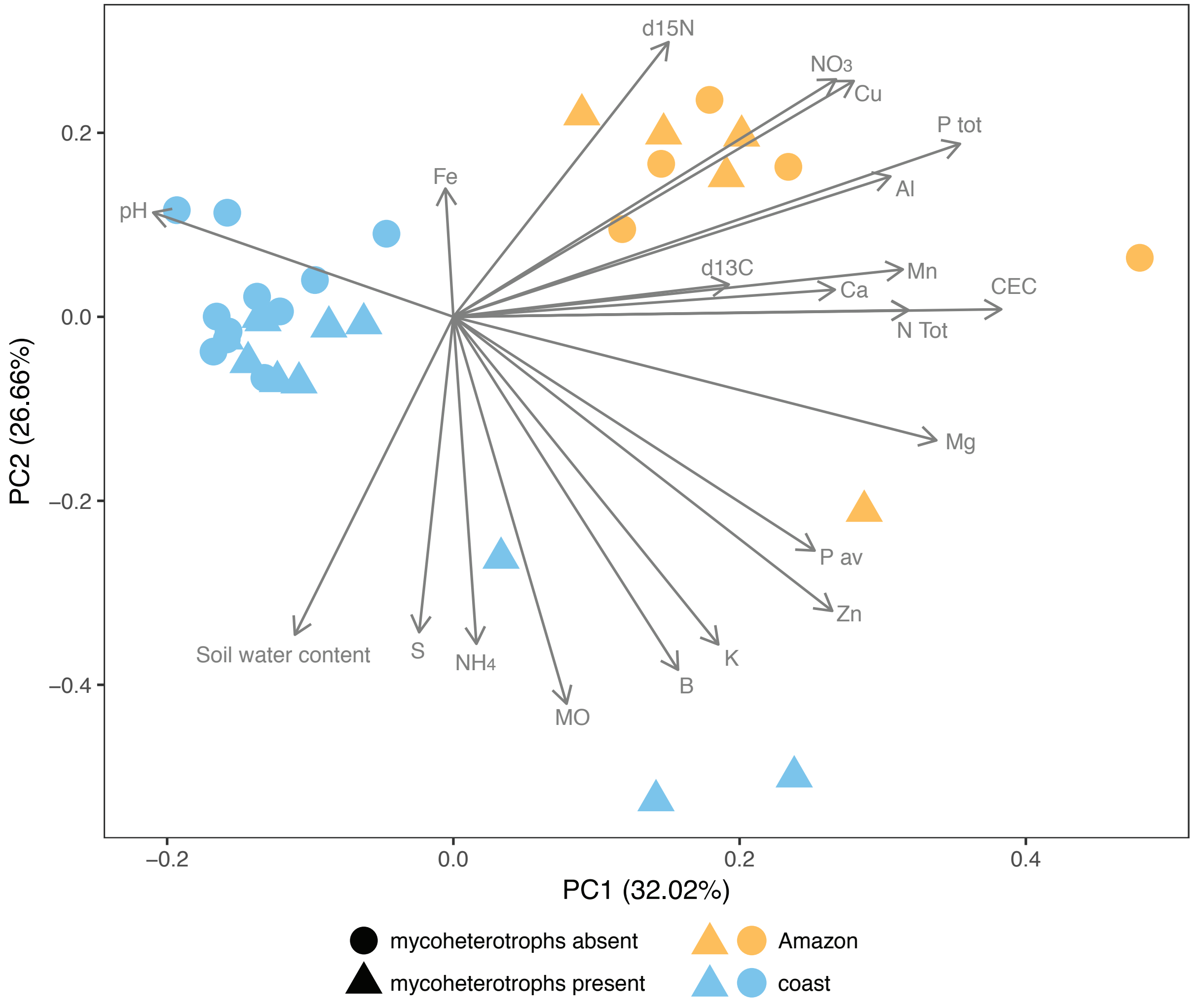

Fig. S1 Principal Components Analysis of the soil properties in the Amazon (yellow) and the Coast (blue). Positive plots (triangles) and negative plots (circles) within each site are represented. Length of the arrows represents the relative importance of predictors.

Table S1 Mycoheterotrophic plant species present in the 16 plots. Number of individuals from each species is presented

		Apteria aphylla	*Gymnosiphon brachychepalus*	*Gymnosiphon divaricatus*	*Sciaphila sp.*	*Sciaphila purpurea*	*Soridium spruceanum*	*Voyria aphylla*	*Voyria chionea*	*Voyria pittieri*	*Voyria tenella*
Coast	**Plot 1**				11						
	**Plot 2**		2	8							6
	**Plot 3**		1	8							
	**Plot 4**	2		8			1	1			
	**Plot 5**		3	7			6				
	**Plot 6**		7	5							
	**Plot 7**						6	8			
	**Plot 8**		4	6				6			3
	**Plot 9**			5			5				
	**Plot 10**			6		4	3				
	**Plot 11**	3	3	4		8	3	1			
Amazon	**Plot 12**										1
	**Plot 13**			3							
	**Plot 14**								1		
	**Plot 15**								1		
	**Plot 16**								2	10	

Table S2: Overall soil parameters in the negative and positive plots within the Amazon and Coast regions.

	Amazon			Coast		
	**Neg plots mean**	**Pos plots mean**	***P***	**Neg plots mean**	**Pos plots mean**	***P***
**pH**	4.26 ± 0.35	4.03 ± 0.18	**0.037***	4.49 ± 0.25	4.31 ± 0.39	0.125
**OM (g/Kg)**	77.77 ± 13.60	93.71 ± 75.14	0.622	88.73 ± 28.06	128.54 ± 47.54	**0.017***
**P total (mg/Kg)**	441.80 ± 73.63	481.40 ± 98.64	0.359	199.82 ± 31.92	212.55 ± 46.13	0.320
**P avail (mg/Kg)**	6.10 ± 0.81	4.85 ± 0.22	**$1.94e^{-05}$***	1.38 ± 0.88	5.21 ± 8.73	0.144
**Ca (cmol/Kg)**	1.68 ± 2.28	0.97 ± 0.94	0.255	0.31 ± 0.09	0.69 ± 0.42	**0.001***
**Mg (cmol/Kg)**	0.64 ± 0.60	0.48 ± 0.24	0.461	0.18 ± 0.05	0.40 ± 0.30	**0.007***
**Al (cmol/Kg)**	2.94 ± 0.63	2.55 ± 0.44	0.256	1.42 ± 0.68	1.39 ± 0.46	0.895
**CEC (cmol/Kg)**	5.26 ± 2.31	3.99 ± 1.42	**0.018***	2.09 ± 0.74	2.89 ± 1.04	**0.004***
**S (mg/Kg)**	47.61 ± 11.04	45.23 ± 7.56	0.689	59.71 ± 14.82	72.80 ± 35.33	0.201
**B (mg/Kg)**	0.65 ± 0.21	1.01 ± 1.39	0.529	0.62 ± 0.18	1.36 ± 1.23	**0.043***
**Zn (mg/Kg)**	4.49 ± 1.50	7.21 ± 4.80	0.205	2.31 ± 1.05	6.03 ± 4.96	**0.011***
**Mn (mg/Kg)**	114.20 ± 125.31	43.66 ± 46.60	0.080	4.10 ± 3.01	16.71 ± 22.39	0.069
**Fe (mg/Kg)**	226.49 ± 150.88	269.41 ± 126.33	0.222	195.04 ± 83.69	201.29 ± 87.90	0.847
**N Total (mg/Kg)**	3366.48 ± 209.92	3854.20 ± 1683.58	0.488	1841.82 ± 395.95	2151.82 ± 373.82	**0.032***
**NH_4 (mg/Kg)**	34.65 ± 15.20	25.40 ± 8.18	0.230	38.90 ± 12.81	48.25 ± 20.43	0.104
**NO_3 (mg/Kg)**	67.99 ± 28.87	46.48 ± 19.91	**0.002***	5.61 ± 2.60	5.33 ± 2.33	0.734
**Soil H_2O c. (g/Kg)**	409.85 ± 29.09	445.25 ± 156.90	0.609	521.84 ± 85.97	654.50 ± 131.04	**0.005***
**K (cmol/Kg)**	0.30 ± 0.04	0.27 ± 0.05	0.116	0.18 ± 0.05	0.42 ± 0.30	**0.011***
**Cu (ppm)**	1.80 ± 0.57	1.87 ± 0.73	0.818	0.55 ± 0.21	0.59 ± 0.20	0.491
**d13C (‰)**	-27.07 ± 5.49	-29.40 ± 0.46	0.307	-29.31± 0.35	-29.27 ± 0.45	0.682
**d15N (‰)**	5.35 ± 0.56	5.33 ± 0.83	0.938	4.30 ± 0.59	3.69 ± 1.11	**0.028***
**N:P**	11.78 ± 6.86	9.72 ± 4.60	0.143	5.07 ± 2.61	3.48 ± 3.05	0.139
**N:K**	241.93 ± 136.82	188.01 ± 114.36	0.082	34.98 ± 24.85	17.82 ± 13.98	**0.046***
**K:P**	0.05 ± 0.003	0.06 ± 0.01	0.130	0.18 ± 0.10	0.18 ± 0.09	0.901
**K:C**	0.004 ± 0.001	0.004 ± 0.001	0.838	0.002 ± 0.001	0.003 ± 001	**0.004***
**C:P**	12.74 ± 1.39	19.30 ± 15.38	0.342	84.94 ± 45.86	70.97 ± 46.27	0.243
**C:N**	1.36 ± 0.70	2.80 ± 3.42	0.328	18.46 ± 7.65	26.57 ± 13.44	0.046

The soil parameters ± SD are significantly different between the negative and positive plots within the respective region at $P < 0.05$ (*). Significant values are represented in bold.

Table S3: Variation of the soil parameters measured in the plots within the Amazon and Coast calculated by the difference between negative and positive plots (deltas).

**Soil parameters**	**Amazon mean Δ**	***P***	**Coast mean Δ**	***P***
pH	0.226	0.278	0.183	0.172
OM (g/Kg)	-15.936	0.790	-39.811	0.074
P total (mg/Kg)	-39.600	0.279	-12.727	0.727
P available (mg/Kg)	1.257	0.900	-3.834	0.161
Ca (cmol/Kg)	0.707	0.105	-0.376	0.223
Mg (cmol/Kg)	0.163	0.482	-0.222	0.081
Al (cmol/Kg)	0.392	0.541	0.032	0.990
CEC (cmol/Kg)	1.262	0.083	-0.798	0.106
S (mg/Kg)	2.378	0.977	-13.095	0.252
B (mg/Kg)	-0.356	0.743	-0.740	0.097
Zn (mg/Kg)	-2.721	0.351	-3.723	**0.031***
Mn (mg/Kg)	70.543	**0.011***	-12.612	0.635
Fe (mg/Kg)	-42.925	0.531	-6.253	0.969
N total (mg/Kg)	487.674	0.402	-310.000	0.442
NH4 (mg/Kg)	9.260	0.459	-9.354	0.199
NO3 (mg/Kg)	21.504	**0.000***	0.278	0.991
Soil water c. (g/Kg)	-35.408	0.846	-132.661	**0.024***
K (cmol/Kg)	0.027	0.966	-0.232	**0.020***
Cu (ppm)	-0.073	0.895	-0.042	0.923
d13C (‰)	2.328	0.116	-0.034	0.999
d15N (‰)	0.026	0.997	0.610	0.053
N:P	2.058	0.316	1.598	0.228
N:K	53.925	0.023*	17.158	0.344
K:P	-0.007	0.961	-0.003	0.982
K:C	0.000	0.928	-0.001	0.158
C:P	-6.560	0.879	13.978	0.311
C:N	-1.434	0.946	-8.116	0.050

The Δ of soil parameters significantly different within the respective site (*: $P < 0.05$) are represented in bold.
